# Should we avoid colleagues in leisure time during the coronavirus disease 2019 (COVID-19) pandemic?

**DOI:** 10.1017/ice.2020.1320

**Published:** 2020-11-13

**Authors:** Lotta-Maria A. H. Oksanen, Enni Sanmark, Sampo A. Oksanen, Veli-Jukka Anttila, Jussi J. Paterno, Lasse Lehtonen, Ahmed Geneid

**Affiliations:** 1 Faculty of Medicine, University of Helsinki, Helsinki, Finland; 2 Department of Otorhinolaryngology and Phoniatrics – Head and Neck Surgery, Helsinki University Hospital, Helsinki, Finland; 3 Nordic Healthcare Group, Helsinki, Finland; 4 Inflammation Center, Helsinki University Hospital, Helsinki, Finland; 5 Department of Ophthalmology, Kuopio University Hospital, Kuopio, Finland; 6 Faculty of Medicine, University of Eastern Finland, Kuopio, Finland; 7 Diagnostic Center, HUSLAB, Helsinki University Hospital, Helsinki, Finland

*To the Editor—* Coronavirus disease 2019 (COVID-19) is a significant occupational threat for healthcare workers (HCWs).^1^ The high number of infected HCWs has been explained with occupational exposure to severe acute respiratory coronavirus virus 2 (SARS-CoV-2). Hospitals have implemented infection control measures including proper personal protective equipment (PPE), universal masking in hospitals, and safety distance between coworkers to reduce the transmission.^2,3^ However, studies of HCW exposure to COVID-19 outside the workplace have not been published.

We examined HCW occupational and nonoccupational exposures to SARS-CoV-2 in tertiary-care hospitals (Uusimaa, Finland) during the first wave of the COVID-19 pandemic through July 15, 2020. Of 1,072 HCWs enrolled in our study, 866 nurses, midwives, and doctors from the Helsinki University Hospital answered questionnaires by July 15, 2020, regarding their infection symptoms, workplace, exposure to COVID-19, and use of PPE. All participants presenting any COVID-19–related symptoms were tested using standard reverse-transcriptase polymerase chain reaction (RT-PCR) methods.^4^ Participants’ medical histories were reviewed in July 2020 for SARS-CoV-2 RT-PCR and antibody results. The demographic backgrounds of the participants were similar to those of Helsinki University Hospital (HUS) personnel overall.^5^

All infected participants were contacted, and the results were confirmed and analyzed. COVID-19 exposure was divided into workplace exposure, outside the workplace exposure, or unknown exposure and further into colleague-related exposure, patient-related exposure, or other exposure. The statistical analysis was performed using the Fisher exact test with SPSS version 27 software (IBM, Armonk, NY).

Overall, 41 participants had COVID-19 and presented either a SARS-CoV-2–positive RT-PCR or antibody test. Of these 41 HCWs, 22 were likely infected at a workplace and 15 were likely infected outside the workplace; the rest remained unclear. The source of infection was likely nonpatient exposure for 21 infected participants (51%). The absolute risk for colleague-origin COVID-19 in this sample was 1.4%, whereas overall regional risk was 0.3%.^5^ Also, 7 workplace-related infections (32%) and 5 infections originating outside the workplace (33%) were transmitted by colleagues.

In our sample, if the source of COVID-19 infection was not a patient, the infection was more likely to be from a colleague (*P* < .001), and 42% of infections from a colleague occurred during leisure time. Notably, participants who treated COVID-19 patients (outside the ICU) had an increased risk for COVID-19 overall (OR, 2.6; 95% CI, 1.4–5.2; *P* = .003), but even then, 5 of 26 COVID-19 cases (19%) were transmitted by colleagues outside the workplace.

Although it may seem self-evident, meeting with other HCWs outside the workplace could introduce an independent risk for a COVID-19 infection, especially among those working with COVID-19 patients. Given the close work communities in health care, where social interactions at work and leisure are traditionally easily mixed, the risk of free-time interactions between workers who work with COVID-19 patients should be carefully considered. In this study, we were unable to provide enough evidence for the authoritative guidance to employees because the sample size was limited. Also, it is likely that some asymptomatic SARS-CoV-2–positive participants were not recognized. Nonetheless, it is imperative to observe social distance in collegial interaction in leisure time until there is an effective vaccine against COVID-19.
